# Editorial: Individual and organizational vulnerability and resilience factors in the COVID-19 pandemic

**DOI:** 10.3389/fpsyg.2023.1216698

**Published:** 2023-09-20

**Authors:** Barbara Juen, Eva-Maria Kern, Sigridur Bjork Thormar

**Affiliations:** ^1^Institute of Psychology, Working Group Emergency Psychology and Psychotraumatology, University of Innsbruck, Innsbruck, Austria; ^2^Faculty of Economic and Organizational Sciences, Chair of Knowledge Management and Business Process Design, Universität der Bundeswehr München, München, Germany; ^3^The Icelandic Centre for Trauma Research and Practice (ICE-TRE), Reykjavík University, Reykjavík, Iceland

**Keywords:** vulnerability, resilience, organizational, pandemic, individual, health care personal

In the beginning the psychological concepts of vulnerability and resilience have been conceptualized as opposing characteristics of individuals. In more recent research vulnerabilities and resilience factors have been treated as characteristics that may be individual, social or organizational (see for example Paton et al., 2001). Furthermore, an individual, group or organization may be characterized by different vulnerability and resilience factors at the same time. Research has also shown that vulnerability is not defined by one characteristic alone because intersectionality is often the care (Ryder and Boone, 2019).

One example for intersectionality is that women are more vulnerable in the COVID 19 pandemic. This finding is important but studies from an intersectional perspective show the additional predictive value of socioeconomic factors, cultural factors and the type of occupation may define how vulnerable or how resilient women can be in a certain social environment (see for example Fordham, 1999). A high number of studies on COVID 19 vulnerabilities show the necessity to focus also on the resilience factors that often accompany potential vulnerabilities. Recent literature about COVID 19 has emphasized specific individual and organizational as well as systemic vulnerabilities that may be characteristic in all pandemics. The same applies for risk factors. The COVID 19 pandemic gives us a chance to further broaden our knowledge about vulnerability and resilience aspects on all levels (individual, social, organizational). For this we need an interdisciplinary and multimethod approach. This has been reached in the given situation because COVID 19 has promoted cooperation and networking between scientists from distant disciplines and origins. With this in mind we collected articles from different disciplinary perspectives: medicine, science, social sciences, public health. Our preferred focus was on mixed method approaches. By analyzing resilience and vulnerability from different angles and on different levels we wanted to gain new insights into the topics.

The following Research Topic of articles gives an overview over different target groups, countries and different perspectives on vulnerability and resilience factors during the pandemic.

Some articles focus on groups that have been emerged as vulnerable during the pandemic like young adults (Kulcar et al.), women and the healthcare and social sector (Riedel et al., 2022a,b). Other articles focus more on the concept of resilience and the factors enabling resilience on each of the above-mentioned levels.

As the studies in this Research Topic show, resilience plays an important role in emergent adulthood. The authors Fu and Wang have shown that for young adults' risk perception of COVID-19 can predict anxiety symptoms. They also showed that quality of life influences both risk perception of COVID-19 and anxiety as a mediator. Resilience on the other hand seems to buffer these effects. A high individual resilience score reduces the effect of risk perception on anxiety. Individual resilience was measured using the CD-RISC by Connor and Davidson (2003). The scale includes five factors: (1) notion of personal competence, high standards and tenacity, (2) trust in one's instincts, tolerance of negative affect, and strengthening effects of stress, (3) positive acceptance of change, and secure relationships, (4) control, and (5) spiritual influences. From these findings the authors conclude that risk communication plays an important role in anxiety management in young adults. At the same time the focus should be on resilience building and quality of life.

Regarding social resources in young adults Kulcar et al. (2022) found that COVID 19 measures heavily influenced young adult's friendships. They experienced major challenges in building new relationships and had difficulties in successfully maintaining existing friendships. As a result, social support by friends diminished, which led to a lack of social resources and loss of resilience. This longitudinal mixed method study could show that the pandemic measures had significant negative effects on friendships for university students.

Talić et al. analyzed resilience and vulnerability factors in students of a military University. Their study investigated individual personality traits (for example extraversion, neuroticism) as well as organizational resilience factors (for example commitment to the organization and satisfaction with study). Furthermore, the researchers investigated health related factors (for example loneliness, quality of life, COVID-19-related stress). Coping strategies were also measured. The authors assumed that coping style would have an influence on stress and psychological wellbeing. The results showed that resilience factors could not predict change in wellbeing over time. But the researchers found some evidence for mediation effects of more active coping styles and the use of social support. Organizational resilience factors plaid a role togethers with personality traits for the wellbeing of the students.

Park et al. used the concept of psychological capital (PsyCap) as a trait influencing both sport community involvement and life satisfaction in Generation Z. Furthermore, the authors referred to the stress process model. Results showed distress modulated the mediation effect of PsyCap especially in Generation Z (Gen Z). Results also showed vulnerability of global sport communities and Gen Z to COVID-19. The authors concluded that support in stress management is of utmost importance for sports fans' community involvement and life satisfaction. Gen Z were more distressed during the pandemic than other participants. Successful stress management was an important prerequisite for the use of community involvement to promote positive resources.

Li and Zhu could show in a Chinese student population that psychological stress had an influence on the students' sense of control as well as on their safety compliance. In this study perceptions of stronger safety regulations enhanced the link between student stress and safety compliance. Future pandemic measures in Universities can profit from these findings.

For young adults we conclude that their dependence of social networks made them especially vulnerable and individual factors like secure relationships and self-reliance as well as active coping but also organizational resilience factors like organizational commitment and community involvement play a role in their wellbeing.

For studies in the healthcare sector we assume that a multilevel approach to resilience is even more important. Panari et al. studied Care Unit identification and perception of safety, as well as personal work engagement in nurses. Their findings show that both aspects seem to be protective against burnout and psychological distress. All interventions done to promote team identification as well as a focus on safety measures for healthcare professionals may positively impact nurses' wellbeing.

A Brazilian researcher team (Pereira-Lima et al.) studied nurses in a low-income country where especially negative effects of the pandemic could be found. Their research showed that dissatisfaction with workplace was rather high and perceived safety very low. Workplace dissatisfaction was significantly linked to emotional exhaustion and depersonalization. Effective support and improvements of workplace safety and quality was seen as crucial for maintaining physical and mental health of nurses in this setting.

Kaltenbrunner et al., an Austrian researcher group, did an interview study with managers in the healthcare sector. Their findings show that Individual personality traits like pragmatism or flexibility and their attitudes like for example optimism are very important for their own resilience as well as the resilience of their teams. Most important was a joint (crisis) understanding between managers and teams expressed for example in a common sense of direction. Furthermore, a focus on social connectedness and a caring attitude were important resilience factors. These attitudes and traits helped to maintain and adapt NPOs' functioning during the pandemic. This study is a good example of a multilevel approach to resilience that emphasizes the interaction of individual and organizational resilience factors.

Regarding healthcare personnel we thus can say that the care orientation of the management is one of the most important resilience factors during the pandemic and that organizational resilience factors play a crucial role in maintaining wellbeing and health of staff (see also Juen et al., 2021; Kreh et al., 2021). This finding might be important also for other sectors in the workforce.

In their study on the French workforce Sandrin et al. examined how a psychological safety climate (PSC) influenced work performance. They analyzed psychological distress and post-traumatic growth during COVID 19 immediately before the second lockdown in France (when cases were steeply rising and vaccination was not yet available). The results show that the safety climate had a positive influence on post-traumatic growth (PTG). Safety climate furthermore influenced work performance and reduced psychological distress. This study shows how important the factor of perceived safety is for wellbeing and performance of healthcare workers during a pandemic.

This study confirms one of the five principles, the principle of safety, that Hobfoll et al. (2007) have named as important resilience factors after emergencies.

Doing a narrative analysis of 48 articles Siller and Aydin analyzed vulnerability and resilience in minority and marginalized individuals and groups: In their view the following three aspects are most important: social inequality must be taken into account because inequality creates vulnerable contexts. In most cases vulnerability has historical roots in the given contexts. In the pandemic these historically grown inequalities lead to special vulnerability factors (communication barriers as well as special risk factors). The authors also assumed that these marginalized and minority groups showed specific resilience during the pandemic. Their results show that this is the case and that a special focus on minority groups and marginalized groups is necessary when looking at disasters. This study is a good example of conceptualizing vulnerability as an integral part of resilience.

We define vulnerability with UNISDR as “*The conditions determined by physical, social, economic and environmental factors or processes, which increase the susceptibility of a community to the impact of hazards*,” (UNISDR, 2015, p. 10). These circumstances are always defined by situation and history. From a Public Health perspective, we see vulnerability as a heightened risk for loss in a crisis situation often including a weakened ability to react in an adequate manner (see also Vaughan and Tinker, 2009). This heightened risk of some population groups in a disaster is closely linked to inequality.

Regarding gender effects of the pandemic we see that for example in a study done by Saloshni and Nithiseelan. In South Africa they studied women workers in vulnerable employment situations for example as domestic help in private households, traders in the informal economy, and small-scale agriculture with no employment contracts or health insurance cover. The study shows the link between socioeconomic and health risks during COVID 19. Although the South African government implemented policies to support workers and reduce the risk faced by vulnerable workers long-term policies aimed at socioeconomic protection are not in place.

A group of Turkish researchers (Demirkaya et al.) analyzed predictors of job quitting during the pandemic and found a significant correlation between depression and work location. The Perceived effect of COVID (PEoC) increased fear, internal and external entrapment, and depression. Also, this study shows the negative effect of life circumstances.

Eckhard et al. presented a measure to assess the psychosocial impact of the SARS-CoV-2 pandemic. The presented measure is based on the International Classification of Functioning, Disability, and Health (ICF) and was developed during the first lockdown in Germany in April 2020. FACT-19 measures stress (pre and post) as well as context factors like barriers and protective factors the authors developed the measure from a former stress barometer a brief screening instrument for emergency situations. The results indicated the suitability of the measure that includes pre-pandemic stress, facilitators and barriers.

Using examples from the COVID 19 pandemic the article Research Topic as a whole is able to show that vulnerability and resilience cannot be treated as opposing concepts. Even people in vulnerable contexts have resilience factors in themselves, in their group and communities as well as in their organizational structures. We can always find resilience and vulnerability factors in any given context. Furthermore, intersectionality plays an important role, vulnerability comes from living in vulnerable circumstances and is closely linked to inequality. And last, we have to always view resilience and vulnerability on the levels of the individual, the social (team/group/community) as well as on the organizational level.

## Author contributions

All authors listed have made a substantial, direct, and intellectual contribution to the work and approved it for publication.
